# Study of the Structure, Oxygen-Transporting Functions, and Ionic Composition of Erythrocytes at Vascular Diseases

**DOI:** 10.1155/2015/973973

**Published:** 2015-10-27

**Authors:** Viktor V. Revin, Natalia V. Gromova, Elvira S. Revina, Natalya A. Mel'nikova, Larisa A. Balykova, Ilia N. Solomadin, Alexander Yu. Tychkov, Nadezhda V. Revina, Oksana Yu. Gromova, Irina V. Anashkina, Viktor A. Yakushkin

**Affiliations:** ^1^FGBOUVPO “N.P. Ogarev Mordovia State University”, Saransk 430005, Russia; ^2^GBUZ RM “National Hospital for War Veterans”, Saransk 430005, Russia

## Abstract

The present paper explores the role of erythrocytes in the pathogenesis of vascular diseases. The state of erythrocytes, their ionic composition and structure, and properties of erythrocytes hemoglobin were studied by using laser interference microscopy, Raman scattering spectroscopy, and capillary electrophoresis. In patients suffering from vascular disorders we identified statistically significant changes in the shape of erythrocytes, their ionic composition, and redistribution of hemoglobin throughout cells.

## 1. Introduction

Vascular diseases (VDs) remain one of the topical problems in medicine. The prevalence rate of vascular disruptions of the brain, caused by hypertension and atherosclerosis of cerebral arteries, in Russia, as well as in most industrialised countries, comprises about 800 cases per 100 thousand people and has no tendency to decline [1, 2]. In the pathogenesis hypoxic ischemic brain damage is synonymous with energy metabolism malfunction, impaired microcirculation due to changes in the rheological properties of blood, the development of edema-swelling of the brain, and activation of Ca-dependent lipid triad with the formation of cytotoxic products. And the main reason is considered to be the structural and functional failure of the endothelium of blood vessels, whereas the role of red blood cells and their oxygen-transporting capacity in the development of vascular diseases remains poorly understood.

In this regard, the purpose of our research was to study the ion composition and structure of erythrocytes as well as hemoglobin content and its capacity to bind oxygen in patients with VD before and after treatment.

## 2. Materials and Methods

The study was carried out with the approval of the Local Ethics Committee under Mordovia State University on the basis of the Mordovian Republican Hospital for War Veterans and is compliant with the principles of Good Clinical Practice. With the informed consent from patients to participate in the study, we have examined 19 women aged from 56 to 73 years (average age 64.3 ± 3.8 years), with transient ischemic attack in the subacute stage of the disease, not previously treated with continuous therapy. Preexisting medical condition for all patients was a combination of primary arterial hypertension of the 2nd degree (code ICD-10: I11) with carotid atherosclerosis (code ICD-10: I70.8). Patients selected for further survey have been suffering from primary arterial hypertension and atherosclerosis of the carotid arteries since the age of 50–57, they did not smoke, nor did they have hereditary abnormalities, and body mass index was 23–29. They were divided into 3 groups depending on the duration of arterial hypertension (group 1 up to 5 years, *n* = 6; group 2 up to 10 years, *n* = 7; group 3 up to 15 years, *n* = 6). The treatment was based on a single procedure and included antihypertensive medication, vasoactive substances, antiaggregants, statins, anxiety medication, antidepressants, neurometabolites, and cholinergic agents. The examination was carried out before and on the 10th day of comprehensive treatment.

The observational group consisted of 10 healthy females of similar age, donors of the republican station of blood transfusion (average age was 58.5 ± 1.9 years), having no VD following the results of preventive examination.

The material of the study was the patients' blood taken on an empty stomach aseptically from the median cubital vein in a volume of 5 mL. Erythrocytes were obtained by centrifuging of whole blood at 1500 g for 15 min.

Determination of potassium, sodium, magnesium, and calcium ions in the erythrocyte mass of human blood was performed by capillary electrophoresis. Erythrocytes were destroyed in a hypotonic solution. Identification and quantification of the ions were performed in the supernatant by indirect absorption at a wavelength of 267 nm on the device Capel-1-5/1-5M (Russia) [3].

Erythrocytes structure and hemoglobin content were determined by method of laser interference microscopy (LIM) on the device MII-4M (Russia) [4, 5].

In contrast to conventional methods of optical microscopy based on the registration of light intensity distribution, the laser interference microscopy allows obtaining the phase distribution in the interference image. LIM operating principle is based on measuring of local phases of the light wave reflected by the object and the reference mirror wave. Overlapping of the reflected wave and the reference mirror wave forms an interference pattern on the object photodetector.

Further, by normalisation of the signal wavelength, we determine the optical path difference of the two waves or phase height (thickness) of the object at a given point and create the phase portrait of a cell that represents the distribution of the phase shift in various areas of the object. The obtained values of phase shift are used to construct a volume (three-dimensional) image of a cell [6].

Study of hemoglobin conformation and properties was carried out using Raman scattering spectroscopy on the device InVia Renishaw (UK) [7, 8]. Raman spectroscopy allows evaluating the state of the cell substances at the level of molecular bonds.

To analyze the changes in hemoporphyrin conformation Raman spectra bands were used (position of the maxima): 1355 and 1375 cm^−1^. These bands show symmetrical vibrations of the pyrrole rings (bonds *С*_*а*_ *С*_*ь*_, C_a_N, and C_a_NC_a_) in molecules of deoxyhemoglobin and hemoglobin associated with ligands, respectively. The ratio of the spectra* I*_1375_/(*I*_1355_ +* I*_1375_) intensities characterizes relative amount of oxyhemoglobin in the blood, the ratio * I*_1355_/*I*_1550_ the relative ability of all hemoglobin in the sample to bind ligands (including oxygen), and the ratio* I*_1375_/*I*_1580_ the relative ability of hemoglobin to secrete ligands. The ratio (*I*_1355_/*I*_1550_)/(*I*_1375_/*I*_1580_) reflects the affinity of hemoglobin for ligands, primarily for oxygen. Ratio* I*_1375_/*I*_1172_ shows the intensity of symmetric and asymmetric vibrations of the pyrrole rings, and its variation may be due to conformational changes of pyrroles [7, 9–11].

Statistical processing of results was carried out with the software package Statistica 8.0 using paired (dependent samples) and unpaired Student's *t*-test, assuming normal distribution of values. Differences at *P* < 0.05 were considered true.

## 3. Results

When examining healthy people we found that the content of potassium, sodium, magnesium, and calcium ions in their erythrocytes was normal [12, 13], (Table 1).

**Figure 1:**
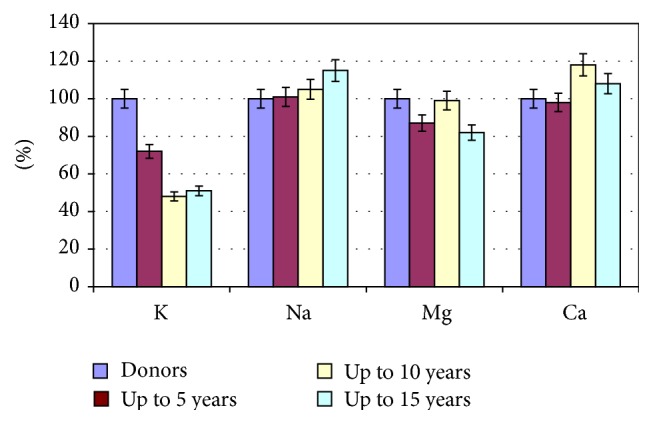
The ionic composition of erythrocytes in patients with VD, depending on the duration of the disease in comparison with healthy women.

We found the ionic imbalance in erythrocytes in the examined patients with VD prior to treatment. So in all patients with VD there was a decreased (in comparison with normal) content of potassium ions, which in 1–3 groups differed from the corresponding control values by 28% (*P* < 0.05), 52% (*P* < 0.01), and 49% (*P* < 0.05), respectively. The content of sodium ions in patients during the first five years of the disease corresponded to the normal range and then increased, exceeding the control values by 16% (*P* < 0.05) in patients of the 3rd group (Figure 1).

Mass concentration of magnesium in erythrocytes of patients of the 1st and 3rd groups was below normal and lower than the corresponding values in the observational group by 13% (*P* < 0.05) and 18% (*P* < 0.05) and the 2nd group was in the normal range. The content of calcium ions in erythrocytes of patients with the minimum and maximum terms of the disease almost corresponded to the norm, whereas in patients of the 2nd group it exceeded normal values and control values by 18% (*P* < 0.05).

**Figure 2:**
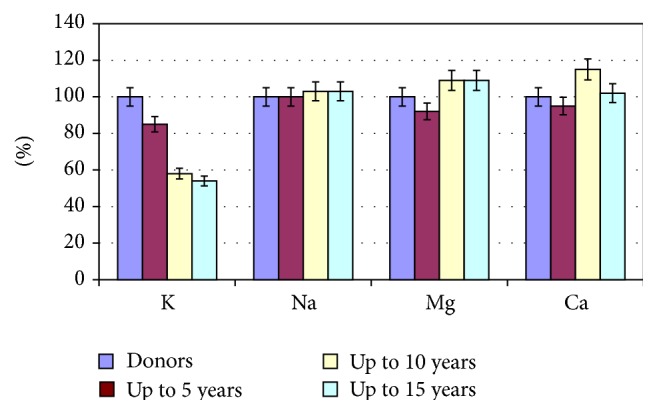
The ionic composition of erythrocytes of patients with cardiovascular diseases after hospital treatment.

Already on the 10th day of treatment we found the stabilisation of the potassium ions in erythrocytes. The concentration of potassium in the 1st and 2nd groups increased by 13% (*P* < 0.05) and 17% (*P* < 0.05) in reference to the original data. However, the level of the electrolyte in erythrocytes in any group of patients did not reach normal. Patients of the 3rd group only showed a tendency towards the increase of potassium ions (Figure 2).

The content of sodium and magnesium ions in erythrocytes of patients with VD after treatment almost corresponded to the norm, and mass concentration of calcium in all groups was at the level of the results obtained prior to treatment, highlighting the need for targeted correction of ion homeostasis (Figure 2).

**Figure 3:**
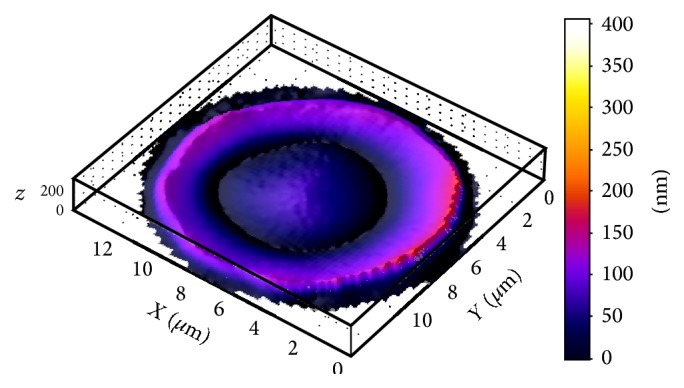
The phase image of human normal erythrocyte obtained using laser interference microscopy.

According to the laser interference microscopy data, area of erythrocytes of practically healthy humans was 181.6 ± 12.6 *μ*m^2^, hemoglobin content was 0.132 ± 0.01 *μ*g, and Hb distribution was uniform throughout the cell volume (Figure 3). The phase image of human erythrocytes is normally characterized by the disk shape flattened in the center and a more rounded edges [14].

In examined patients with VD the area of erythrocytes and the content of hemoglobin differed from control values.

**Figure 4:**
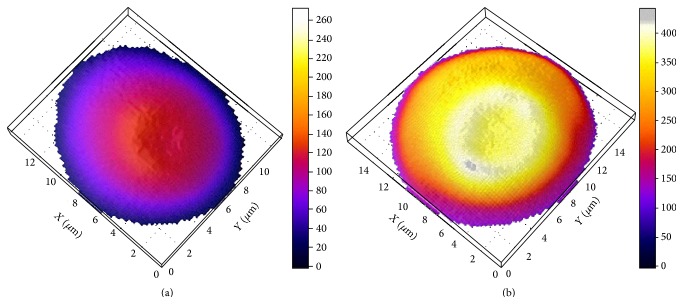
The phase image of human erythrocyte before the treatment obtained using laser interference microscopy. Duration of the disease: (a), up to 5 years; (b), up to 15 years.

In patients with a short period of the disease the erythrocytes with flattened shape were observed (Figure 4(a)). In patients with prolonged course of the disease the erythrocytes form was close to spherical, hemoglobin not evenly distributed throughout the volume of the cell (Figure 4(b)).

**Figure 5:**
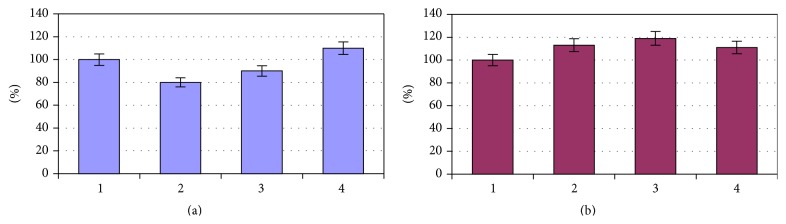
The erythrocytes area (a) and hemoglobin content (b) in patients with vascular diseases depending on the disease duration: 1, donors; 2, period of the disease up to 5 years; 3, period of the disease up to 10 years; 4, period of the disease up to 15 years.

In patients of the 1st group, the area of erythrocytes decreased by 20% compared with control values (*P* < 0.05) and hemoglobin content was exceeding the control level by 13% (*P* < 0.05) (Figure 5). The area of erythrocytes in patients of the 2nd and 3rd groups was almost on the same level as the control values, and the hemoglobin content was higher than values of healthy individuals by 19% (*P* < 0.05) and 11% (*P* < 0.05), respectively (Figure 5).

**Figure 6:**
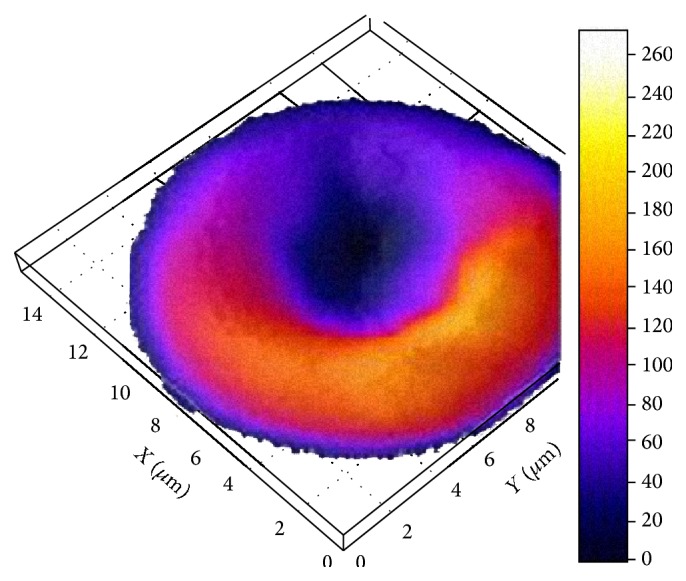
Image of human erythrocyte after treatment obtained using laser interference microscopy.

On the 10th day of therapy, the shape of erythrocytes among the patients of all groups became more discoid than before treatment, and the hemoglobin was distributed more evenly throughout the volume of the cell. At this the spatial relation between the central part of the erythrocyte and its periphery was still different from the control group (Figure 6).

**Figure 7:**
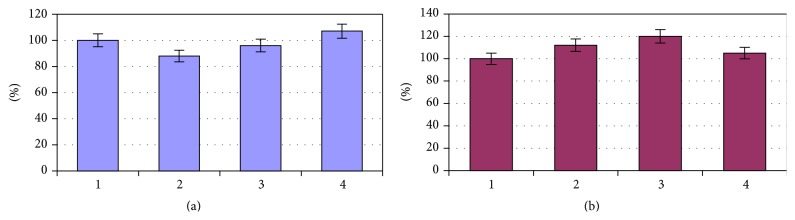
The erythrocytes area (a) and hemoglobin content (b) in patients with vascular diseases after a course of treatment depending on the disease duration: 1, donors; 2, period of the disease up to 5 years; 3, period of the disease up to 10 years; 4, period of the disease up to 15 years.

On the 10th day of treatment in patients of the 2nd and 3rd groups the area of the red blood cells reached the norm, and the hemoglobin content was almost unchanged compared with the original data (Figure 7).

Properties of hemoglobin's hemoporphyrin were investigated by Raman spectroscopy. Analysis of the ratio of Raman spectra bands intensities showed that cardiovascular diseases are accompanied by an increase of the o-Hb amount in all the groups by 26% (*P* < 0.05), 20% (*P* < 0.05), and 24% (*P* < 0.05), respectively. In turn, the ability of hemoglobin to bind oxygen is reduced by 41% (*P* < 0.01), 39% (*P* < 0.01), and 26% (*P* < 0.05) compared to control data.

When studying the relationship of the band intensities* I*_1375_/*I*_1580_ of hemoglobin spectrum (the ability of hemoglobin to release ligands) it is shown that this indicator among all patients with VD is at the level of the control values.

The hemoglobin oxygen affinity was reduced by 34% (*P* < 0.05) and 30% (*P* < 0.05) in patients in groups 1 and 2, respectively, and had a tendency towards decreasing in the 3rd group in reference to the control level. The relation of bands intensities* I*_1375_/*I*_1172_ characterizing the evidence of symmetric and asymmetric stretch of pyrrole rings had the tendency towards increasing in comparison with the control values in the 1st and 3rd groups of patients and was statistically significantly (57%, *P* < 0.05) higher in the 2nd group (Table 2).

In the course of treatment we did not reveal significant differences in the spectral characteristics, reflecting the oxygen-transport function of hemoglobin in relation to the original data, which indicates the absence of the influence of traditional therapy on functional properties of hemoglobin (Table 2).

**Figure 8:**
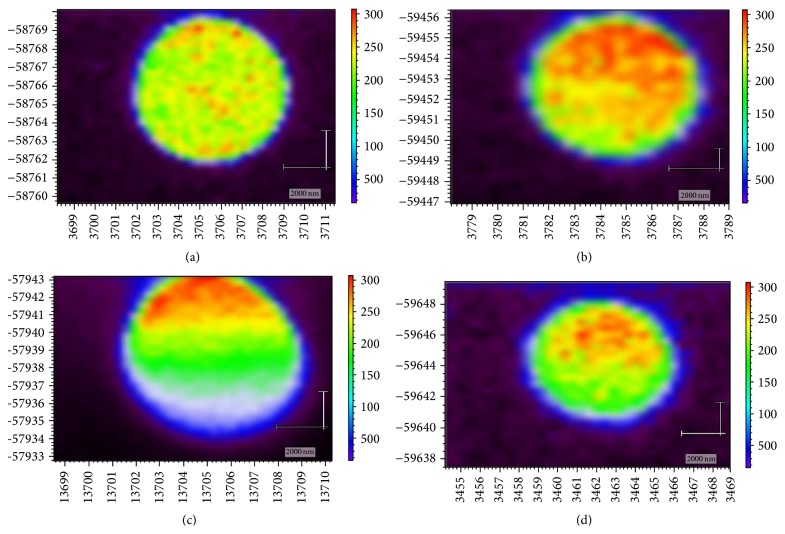
Image of human erythrocytes: (a), control group; (b), early stage of the disease; (c), late stage of the disease; (d), after treatment (red, the most intense zone of Raman response up to 200 rel. units; green, area with an intensity up to 150 rel. units; blue, the substrate (the glass in this case)).

On the basis of the obtained spectra and their intensities, Raman images of the erythrocytes of the control group people and CVD patients were obtained using the software package WIRE 3.3 (Figure 8).

The image nature indicates substantial hemoglobin redistribution in the erythrocyte volume of CVD patients. Therapy on the early stages of the disease led to a partial recovery of hemoglobin spatial distribution in erythrocytes.

## 4. Discussion

Change in the ionic composition of cells is a factor which plays a significant role in the conformational rearrangements of membrane proteins and lipids, in the erythrocyte cytoskeleton change, and hence in a change of their structure and hemoglobin redistribution in the cytoplasm [15].

Identified changes in the ionic composition of the erythrocytes in patients with CVD may lead to disruption of the membrane structure and initiate a chain of interconnected destructive reactions, including in the erythrocytes' hemoglobin.

Studying hemoglobin properties in CVD patients before and after treatment showed the presence of such transformations.

It is known that, during formation of hemoglobin complexes with ligands, namely, O_2_, iron turns into low-spin state, wherein Fe^2+^ size is less than it was in deoxyhemoglobin and iron atom is practically in a cavity of the porphyrin ring, increasing the porphyrin diameter, thereby changing conformation of the molecule as a whole [16, 17].

Disturbance of the spatial relationship between the porphyrin ring, iron and oxygen changes the strength of their interaction. This leads to disruption of oxygen-transport function of hemoglobin. We assume that a possible cause of change in the conformation of hematoporphyrin of hemoglobin may be a disruption of the ion balance between the cell and the extracellular environment.

Moreover, the change in the ionic composition of erythrocytes entails deterioration in their deformability and the increase of the aggregation capacity [18, 19]. This is explained by the fact that the flexible mobility of erythrocyte membranes is based on the binding of calcium and magnesium with spectrin (the main protein of the cytoskeleton) [20]. Changes in the cytoskeleton of erythrocytes can cause irregularities in the distribution of hemoglobin which are either membrane-bound or cytosolic [8, 12].

It is obvious that changes in the conformation of the hemoglobin of erythrocytes disrupt its ability to bind and release oxygen, which can lead to incomplete release of oxygen from oxyhemoglobin, its insufficient penetration to tissues, and progression of tissue hypoxia among patients with VD.

The decrease in the deformability of erythrocyte membrane also leads to poor oxygen exchange [21].

Perhaps it is the hypoxia that may underlie endothelial dysfunction, a primary stage of pathogenesis of vascular disorders, and the impact on this stage of the disease will let us improve the results of treatment of patients with VD. In addition, the values of the ionic composition of red blood cells and oxygen-transporting properties of hemoglobin may be early markers for the development and progression of vascular diseases.

## 5. Conclusion

We have found that VDs are accompanied by pathological changes of the red blood cells, primarily structural and physiological rearrangements of the hemoglobin of erythrocytes, the main transporter of oxygen in the body. Along with the changes in the structure we observed disorders in the distribution of ions of potassium, sodium, magnesium, and calcium and the decreased ability of hemoglobin to bind oxygen in VD patients compared with healthy people. These data suggest that high mortality in vascular diseases may be attributed to disorders of the blood vessels as well as to changes in the structure and functions of erythrocytes.

The lack of significant changes in the oxygen-binding ability of the hemoglobin of erythrocytes under the accepted treatment regimen may evidence the appropriate use of additional medication instrumental in enhancing oxygen-binding function of erythrocytes and compensating for the lack of oxygen in organs and tissues of the body.

## Figures and Tables

**Table 1:** Ionic composition of erythrocytes (maximum ± minimum) in healthy donors (observational group, *n* = 10).

Group	Indicators			
	К^+^, mg/L	Na^+^, mg/L	Mg^2+^, mg/L	Са^2+^, mg/L
Control	3210.4 ± 41.7	753.0 ± 23.2	103.0 ± 4.0	22.4 ± 1.3
Normal values	3003.0–5460.0	207.0–644.0	39.6–108.0	9.0–28.0

**Table 2:** Changes of oxygen-transport capacity of hemoglobin during treatment (maximum ± minimum) among patients with VD and healthy persons.

Variants	Rel. amount of о-Hb in blood, rel. un. *I*_1375_/(*I*_1355_ + *I*_1375_)	Hb ability to bind ligands (including O_2_), rel. un. *I*_1355_/*I*_1550_	Hb ability to secrete ligands, rel. un. *I*_1375_/*I*_1580_	Hb affinity to O_2_, rel. un. (*I*_1355_/*I*_1550_)/(*I*_1375_/*I*_1580_)	Intensity of sym. and asym. stretch of pyrrole rings, rel. un. *I*_1375_/*I*_1172_
Control	0.54 ± 0.05	0.90 ± 0.17	0.69 ± 0.04	1.30 ± 0.09	0.94 ± 0.11

Patients before treatment					
Group 1	0.68 ± 0.06^*∗*^	0.53 ± 0.09^*∗∗*^	0.63 ± 0.06	0.86 ± 0.11^*∗*^	1.25 ± 0.13
Group 2	0.65 ± 0.06^*∗*^	0.55 ± 0.11^*∗∗*^	0.61 ± 0.02	0.91 ± 0.09^*∗*^	1.48 ± 0.15^*∗*^
Group 3	0.67 ± 0.05^*∗*^	0.67 ± 0.07^*∗*^	0.68 ± 0.10	0.99 ± 0.12	1.23 ± 0.11

Patients after treatment					
Group 1	0.68 ± 0.07^*∗*^	0.55 ± 0.08^*∗∗*^	0.65 ± 0.09	0.85 ± 0.09^*∗∗*^	1.16 ± 0.10
Group 2	0.59 ± 0.04	1.02 ± 0.08	0.68 ± 0.11	1.47 ± 0.11	1.28 ± 0.11^*∗*^
Group 3	0.65 ± 0.04^*∗*^	0.58 ± 0.02^*∗∗*^	0.65 ± 0.13	0.92 ± 0.13^*∗*^	1.29 ± 0.14^*∗*^

Note: ^*∗*^ *P* < 0.05; ^*∗∗*^ *P* < 0.01 compared to control.
